# Analysis of Prefrontal Single-Channel EEG Data for Portable Auditory ERP-Based Brain–Computer Interfaces

**DOI:** 10.3389/fnhum.2019.00250

**Published:** 2019-07-25

**Authors:** Mikito Ogino, Suguru Kanoga, Masatane Muto, Yasue Mitsukura

**Affiliations:** ^1^Dentsu ScienceJam Inc., Tokyo, Japan; ^2^National Institute of Advanced Industrial Science and Technology, Tokyo, Japan; ^3^WITH ALS General Incorporated Foundation, Tokyo, Japan; ^4^School of Integrated Design Engineering, Keio University, Kanagawa, Japan

**Keywords:** electroencephalogram, brain–computer interface, auditory event-related potential, single-channel data, portable measurement device

## Abstract

An electroencephalogram (EEG)-based brain-computer interface (BCI) is a tool to non-invasively control computers by translating the electrical activity of the brain. This technology has the potential to provide patients who have severe generalized myopathy, such as those suffering from amyotrophic lateral sclerosis (ALS), with the ability to communicate. Recently, auditory oddball paradigms have been developed to implement more practical event-related potential (ERP)-based BCIs because they can operate without ocular activities. These paradigms generally make use of clinical (over 16-channel) EEG devices and natural sound stimuli to maintain the user's motivation during the BCI operation; however, most ALS patients who have taken part in auditory ERP-based BCIs tend to complain about the following factors: (i) total device cost and (ii) setup time. The development of a portable auditory ERP-based BCI could overcome considerable obstacles that prevent the use of this technology in communication in everyday life. To address this issue, we analyzed prefrontal single-channel EEG data acquired from a consumer-grade single-channel EEG device using a natural sound-based auditory oddball paradigm. In our experiments, EEG data was gathered from nine healthy subjects and one ALS patient. The performance of auditory ERP-based BCI was quantified under an offline condition and two online conditions. The offline analysis indicated that our paradigm maintained a high level of detection accuracy (%) and ITR (bits/min) across all subjects through a cross-validation procedure (for five commands: 70.0 ± 16.1 and 1.29 ± 0.93, for four commands: 73.8 ± 14.2 and 1.16 ± 0.78, for three commands: 78.7 ± 11.8 and 0.95 ± 0.61, and for two commands: 85.7 ± 8.6 and 0.63 ± 0.38). Furthermore, the first online analysis demonstrated that our paradigm also achieved high performance for new data in an online data acquisition stream (for three commands: 80.0 ± 19.4 and 1.16 ± 0.83). The second online analysis measured online performances on the different day of offline and first online analyses on a different day (for three commands: 62.5 ± 14.3 and 0.43 ± 0.36). These results indicate that prefrontal single-channel EEGs have the potential to contribute to the development of a user-friendly portable auditory ERP-based BCI.

## 1. Introduction

Brain-computer interface (BCI) is a popular technology related to the electroencephalogram (EEG). This non-invasive technology has the potential to provide patients suffering from severe communication disorders with the ability to communicate through translation of specific electrical patterns in the brain into predefined commands. The development of BCI technologies has allowed patients with amyotrophic lateral sclerosis (ALS) to communicate with others without the requirement of body movement (Sellers and Donchin, 2006; McFarland et al., 2011; Spataro et al., 2017). Although these developments have made the realization of BCI technologies possible in laboratory environments, many ALS patients desire the use of these technologies in their daily lives, in their family and in their home environments.

Traditional EEG devices make use of gel-based multi-electrodes (over 16 channels) for data acquisition. This equipment is difficult for untrained individuals (such as family and helpers) to use because a high level of expertise is needed to accurately attach the electrodes while properly adjusting the quality of the acquired signals. The family/helper has to learn how to appropriately position the electrodes as well as how to correctly adhere the electrodes to the head if they are to operate BCIs without significantly worsening their performance. To alleviate the burden of mastering such complex skills for the family/helper and to minimize the stress imposed on the patient during the preparation of the device, the number of electrodes should be decreased, and the equipment should be simplified as much as possible. Recent developments in EEG devices have made it possible to decrease the number of required electrodes and achieved simple data acquisition by integrating a wireless communication module and dry electrodes (Rogers et al., 2016; Krigolson et al., 2017; Minguillon et al., 2017). These devices have the potential to provide patients with easy-to-use EEG-based BCIs.

An event-related potential (ERP) is a neuronal response to a rare, though already known, event. ERPs have been widely used in BCI paradigms with ALS patients (Hill et al., 2005; Höhne et al., 2010; Schreuder et al., 2011). Visual or auditory stimuli are often employed as the events to elicit the specific electrical response. These responses can be observed in EEGs, and the paradigm only requires concentration during a period in which one assesses the rarity of the event. Even if ALS patients enter a “completely-locked-in” or “totally-locked-in” state (CLIS or TLIS) accompanied by a loss of visual function due to excessive dryness of the retina caused by muscle dysfunction associated with the eyelid, their auditory functions fortunately remain intact. On account of this situation, some studies have recently explored incorporating auditory stimuli into a BCI paradigm with the goal of developing generalized ERP-based BCI technologies for ALS patients (Hill et al., 2014; Kaongoen and Jo, 2017; Hammer et al., 2018).

To obtain a high level of BCI performance and usability, selection of effective auditory stimuli is important to induce strong ERPs and to maintain a high level of participant's motivation during its operation. A synthetic sound group composed of different pitches and different arrival directions is often presented to the user during auditory ERP-based BCI paradigms. However, the repetitive unnatural sounds can easily make the user tired, thereby significantly decreasing their motivation. To avoid such situations during daily use, natural sounds have been used in auditory ERP-based BCI paradigms. Simon et al. (2015) proposed a multi-command auditory ERP-based BCI that makes use of five natural sounds (a duck, singing bird, frog, seagull, and a dove) presented from five different directions (left, center-left, center, center-right, and right) in the paradigm. It was demonstrated that the natural sounds improve the performance of auditory ERP-based BCI, presumably because natural sounds better serve to maintain the motivation of participants until the end of the period. Besides, the differences are easier to distinguish. A further study by Huang et al. (2018) reported that natural sounds influenced subjective motivation and improved the performance of auditory ERP-based BCI. Until now, the mean offline detection accuracy of auditory ERP-based BCI paradigms using traditional EEG devices has reached 80.0% (Baykara et al., 2016; Heo et al., 2017), which exceeds the accuracy required (70.0%) for satisfactory communication in BCI technologies (Käthner et al., 2013).

Whereas previous studies used traditional EEG devices for data acquisition and ERP detection, the usability of the EEG device in daily life must be taken into consideration to make it useful in practical terms for patients. In addition, the detection accuracy of auditory ERP-based BCI paradigms utilizing a recently developed EEG device that makes use of only a prefrontal single-channel measurement electrode, is not clear. Therefore, analyzing a portable auditory ERP-based BCI is still an important challenge to overcome in complicated traditional measurement environments. The aim of this study is to analyze auditory ERP waveforms acquired from a consumer-grade single-channel EEG device and to clarify the detection performance under offline and online conditions.

This paper is organized as follows: section 2 describes the specifics of the EEG recordings, such as subject characteristics and auditory stimuli explanations. Additionally, the methods for capturing and classifying the EEG data in offline and online processing are described, section 3 presents the results of analyzing the auditory ERP waveforms. Finally, section 4 discusses the results and presents conclusions regarding the plausibility of applying auditory ERP-based BCI using only prefrontal single-channel EEG in daily-life communication.

## 2. Materials and Methods

### 2.1. Participants

Nine healthy participants without any myopathy (three females, age: 20-50, mean: 33.0, SD: 8.2) and one ALS patient (a 31-year-old male) took part in the experiments. The outbreak of his ALS occurred ~4 years prior to this experiment and his ALS-functional rating scale score, as obtained on the day of the EEG recordings, was 17. Before the experiments, all participants provided written informed consent and were asked to abstain entirely from intake of caffeine, nicotine, and alcohol after 9 pm on the previous day.

### 2.2. EEG Recordings

To implement an easy-to-use EEG-based BCI, we used a consumer-grade mobile EEG device called MindWave Mobile with a BMD chipset (NeuroSky Inc.) This device has a measurement electrode and a reference electrode for fixed locations Fpz and A1 according to the international 10–20 system (Jasper, 1958). The reason why we used the device is that an auditory ERP response can be detected even from the frontal area (Holm et al., 2006; Höhne and Tangermann, 2014; Käthner et al., 2015). Furthermore, the signal quality from the frontal area is better than that of other locations as the location has less influence on air contamination between the electrode and the scalp. We therefore used this device to implement a portable ERP-based BCI. The sampling rate of this device is 512 Hz for data acquisition. The device installs a Bluetooth module for wireless communication between itself and a storage device. This module removes the coupling cables for the amplifier and recorder; this helps to avoid movement constraints caused by the length of the cables, such as sitting on a chair that is close to the amplifier and recorder. A notch filter was applied to the EEGs to attenuate the 50 Hz power-line noise. EEG recordings of healthy subjects were acquired in the same room. EEG recordings of the ALS patient were acquired in his home. Each subject was asked to sit on a chair/wheelchair (WHILL Model C, WHILL, Inc.) with their eyes closed and unmoving, and was asked to put on a canal-type earphone (EM01K, FOSTEX) that was physically connected to an iPad (CPU: Apple A9, RAM: 2 GB, and OS version: 11.4.1).

### 2.3. Auditory Stimuli

**Figure 1:**
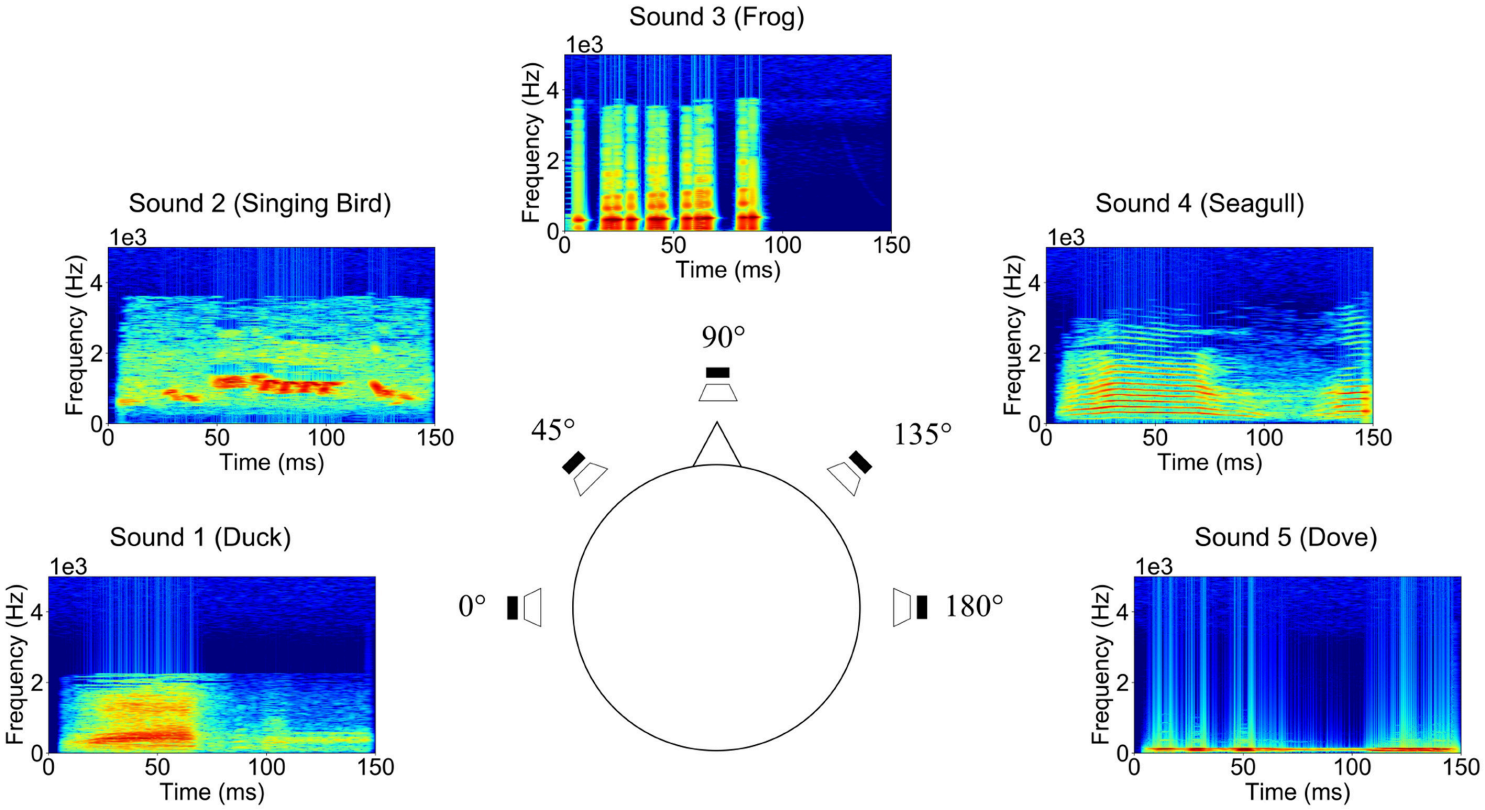
Spectrograms and directions of the five natural sounds (0°: duck, 45°: singing bird, 90°: frog, 135°: seagull, and 180°: dove) presented in our experiments.

In this study, we chose five natural sounds used in a previous study (Simon et al., 2015): (i) duck, (ii) singing bird, (iii) frog, (iv) seagull, and (v) dove, which include several spectral structures and temporal changes. To assist in the discrimination of sounds, each sound came from a different direction, as conveyed through differential use of the earphones: (i) left, (ii) center-left, (iii) center, (iv) center-right, and (v) right. The spectrograms and directions of these sounds are shown in Figure 1. The sounds were downloaded from the webpage http://bbcsfx.acropolis.org.uk/. Time length and directions were modified.

The sound directions were synthetically generated with Python code in accordance with a previous study (Käthner et al., 2013). The interaural level difference (ILD) and interaural time difference (ITD) were adjusted. For the sound of 0° (left), 90° (center), and 180° (right), we only adjusted ILD to render sound directions. The ITD was applied for sounds located at 45° (center-left) and 135° (center-right). That metric can be obtained from the following equation:

(1) $$\begin{array}{r} \text{ITD}=\frac{r}{c}\left(\theta +\sin\left(\theta \right)\right), \end{array}$$

where *r* is the radius of the human head, *c* is the speed of sound, and θ is the azimuth of the sound source localization. We set *r* = 0.9, *c* = 340.0, and θ = {45°, 135°} to synthesize the sound directions. The impressions of 45° and 135° were improved by the subjectively selected ILD (Käthner et al., 2013). To prevent the participants from listening to specific sounds, the volume of all sounds was normalized.

### 2.4. Experimental Design

The experimental design was approved by the Ethical Review Committee of Dentsu ScienceJam Inc. with approval number 006 in October 2018. The experiments consisted of an offline analysis and online analysis. First, we presented all kinds of natural sounds (i.e., duck, singing bird, frog, seagull, and dove) at least five times for each participant as a calibration phase at the beginning of the experiments to ensure that the participants recognized the sounds easily. The sound volume level was adjusted during listening. If he/she requested to hear any of the sounds again, we allowed him/her to listen to the sounds once again.

**Figure 2:**
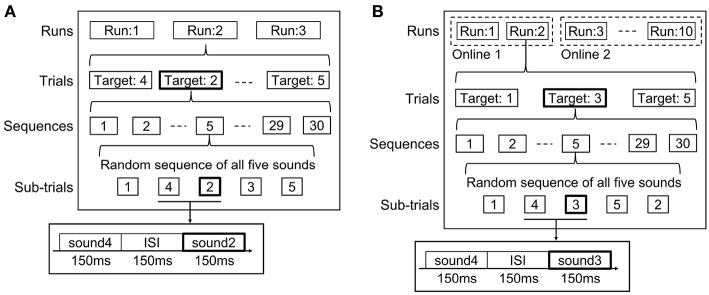
Experimental protocols for one participant in **(A)** offline analysis and **(B)** online analyses. Two runs with online data were performed on the same day as the offline analysis (online experiment 1). The other eight runs were conducted 6 months later (online experiment 2). Five natural sounds were randomly presented, and the participants mentally counted the presence of the target sound during EEG recordings.

Figure 2A shows the experimental protocol for the offline analysis. During the experiments, three runs were conducted for each participant. Each run consisted of five trials. At the start of each trial, one of the five sounds was randomly assigned to be the target sound. This assignment continued for the duration of the trial. Over the five trials, each of the five sounds was selected as the target sound only once. Each trial was composed of 30 sequences. Each sequence contained a random permutation of five sounds (five sub-trials). In brief, one trial contained 30 sub-trials for ERP waveforms caused by one target sound, and four types of 30 sub-trials for non-ERP waveforms based on four non-target sounds. In addition, we were able to, respectively, obtain five grand-average ERP waveforms across the 30 sub-trials for five sound labels from one trial. The participant was asked to focus on the target sound and mentally count the appearance of the targets throughout a trial. We also requested the participants to count from one to 10 three times rather than counting to 30 because numbers >10 impose a higher mental load and can contribute to participant fatigue. Participants were given a rest between runs and decided the duration of their rest each time because a fixed-rest duration affects motivation in BCI tasks (Huang et al., 2018). Note that no participant chose to rest over 10 min.

In a previous BCI study, Simon et al., 2015 set the sound duration to 150 ms, the interstimulus interval (ISI) to 250 ms, and the number of sequences to 15. According to this study, we set the sound duration in our experiment to 150 ms. On the other hand, we shortened the ISI to 150 ms and increased the number of sequences to 30 because we could not confirm clear grand-average ERP waveforms and sufficient accuracy during the pre-testing phase in which we used 250 ms ISI and 15 sequences with the consumer-grade EEG device. Hence, we increased the number of sequences to 30. In the case of using 250 ms ISI and 30 sequences, the total length of one trial became 1 min, which was too long for participants to concentrate on counting the sound stimuli, thereby leading to low accuracy. Therefore, we set the ISI to 150 ms, shortened the length of one trial, and used 30 sequences, simultaneously. Such short ISI was also used in Höhne et al. (2011) and is common in visual ERP-based BCIs (Käthner et al., 2015; Hammer et al., 2018).

To avoid the effects of artifacts caused by body or ocular movements during the offline analysis, we visually inspected whether the data included artifacts after each trial. We set upper and lower bounds to 40.0 and −25.0 μV for the identification. When artifact peaks exceeding the thresholds were present in the recorded EEG dataset, the trial was conducted again to collect the predefined number of trials. In the online experiment, the thresholds automatically detected the artifacts. Consequently, the iPad application indicated to the subjects to record the trial once again. In addition, to avoid any changes to device configuration (such as reattachment of the device that may lead to deterioration of the constructed classifiers), we continuously recorded and tested EEG data under offline and online conditions for ~1 h. In the online experiments, we reduced the number of commands (types of target sounds) from five to three to keep participants motivated. In the pre-test trial, we tested the detection performance of five commands. However, their accuracy was not high enough to keep participants' motivated, and they tended to report fatigue or boredom. Therefore, we reduced the number of target commands to keep the motivation in the online experiments.

Sound 2 (singing bird) and sound 4 (seagull) were not used as target sounds in the online experiments. The previous studies used the sound localization of 0°, 90°, and 180° for auditory BCIs (Höhne et al., 2011; Lopez-Gordo et al., 2012; Huang et al., 2018), because the direction confusion was stronger when the directions of 45° and 135° were used (Schreuder et al., 2010). Hence, we selected 0°, 90°, and 180° for the three target sounds (duck, frog, and dove) and evaluated detection performance with three commands in the online analysis. However, all kinds of sounds were presented in the experiments; thus, sounds 2 and 4 were always assigned to non-target sounds. Ten runs were used for evaluation in the online analysis (Figure 2B). Two runs were obtained on the same day as the offline analysis (online experiment 1) because setting this number of runs also depended on fatigue and tiredness, reported after more than 1 h of recording in pre-test trials. Another eight runs were conducted 6 months later (online experiment 2). All data obtained in the offline experiment were used for the online experiment 1 as learning data. For online experiment 2, two runs were conducted before the experiment and added to the learning data. All three sounds that arrived from left, center, and right directions were used as the target sound two times each. After each trial, the acquired 150 sub-trial data were separated into five groups based on the presented sound labels, and then they were, respectively, averaged over 30 epochs to obtain grand-average undefined (ERP/non-ERP) waveforms. The grand-average waveform that presented the highest probability of the target sound among the five waveforms was detected as ERP waveform. If the waveform of sound 2 or 4 had the highest probability of the target sound, another waveform having the second highest probability was detected as ERP waveform. After the detection, the number corresponding to the predicted sound was visually presented on the iPad display to maintain the participant's motivation (Nijboer et al., 2008). After each trial, participants opened their eyes (before closing them again) to check the obtained number.

### 2.5. Feature Selection and Classifier

We applied a fourth-order band-pass Butterworth filter with 0.5 Hz and 10 Hz cut-off frequencies to each sub-trial data (epoch). One-second epochs (512 samples) after the onsets of auditory stimuli were extracted for both analyses. Each extracted epoch was baseline-corrected by the average value of pre-stimulus data within an interval of 100 ms (Simon et al., 2015). The baseline-corrected epochs were consequently down sampled to 16 Hz and used as the features for classification; therefore, the dimensionality of features was 16. Each grand-average waveform was calculated before this down sampling process.

For feature selection and ERP detection, we used a stepwise linear discriminant analysis (SWLDA), which has been widely used in auditory ERP-based BCI studies (Simon et al., 2015; Onishi et al., 2017). The SWLDA learns two-class (target and non-target) information using the training data that contains all sound labels with two patterns (e.g., EEG features while presenting sound 1 as the target in trial 1 of run 2, and EEG features while presenting sound 1 as the non-target in trial 2 of run 3), and then predicts the probability of target for the testing data based on the trained SWLDA classifier. To express the variance of data, the training data remains sub-trial information; however, the testing data consists of grand-average undefined (ERP/non-ERP) waveforms over 30 sub-trials in one trial for the respective sound labels. This ensures a high detection accuracy considering the situation of actual BCI operations where users do not allow poor and vulnerable BCIs. SWLDA selects important feature variables automatically with a combination of forward and backward steps (Krusienski et al., 2006). First, *p*-values are calculated with the least-squares regression for feature variables. The feature variable set, which has a lower *p*-value than the input threshold (*p*-value < 0.10), is pooled into the linear discriminant analysis (LDA) function. A new feature variable is continuously added to the LDA function. If the feature variables are no longer significant after updating *p*-values, they are removed from the feature variable set (*p*-value > 0.15). This process is repeated until no additional feature variables meet the input or output criteria.

### 2.6. Assessment

A key factor for ERP-based BCIs is the detection accuracy of target and non-target ERPs. We separately calculated the accuracy for each subject. In the offline analysis, the accuracy was calculated through a leave-one-trial-out cross-validation (LOTOCV) procedure. The data from one trial was used for testing, while that of the others was used for training. Thus, 2,100 sub-trials (14 trials × 30 sequences × 5 sub-trials) constituted the training data and five grand-average ERP/non-ERP waveforms (150 sub-trials) were the testing data in each validation step in a subject. In the online analysis, all data obtained in the experiments were grand averaged for the testing data. Furthermore, the detection performance was calculated by applying the selected feature variables, and their coefficients in the LDA function optimized by all sub-trial data in the offline analysis.

In this paper, the evaluation metric “Accuracy" has the same meaning as the true positive rate (TPR) in a confusion matrix. The matrix collects the decisions of target and non-target sounds based on posterior probabilities of SWLDA. TPR refers to how accurately EEG recordings were classified as targets during the listening of the target sounds; therefore, TPR is an effective metric for evaluating ERP-based BCI performance.

Apart from detection accuracy, other evaluation metrics may be used for BCI systems. Previous studies have used the information transfer rate (ITR), which has the advantage of incorporating both speed and accuracy into a single value (Cheng et al., 2002; Wolpaw et al., 2002; Wang et al., 2017). It indicates the amount of information communicated per unit of time. The ITR can be calculated using the following equation:

(2) $$\begin{array}{r} \text{ITR}=\left(\log_{2}N+P\log_{2}P+\left(1-P\right)\log_{2}\frac{1-P}{N-1}\right)*60/T, \end{array}$$

where *N* is the number of selectable commands (*N* = {2, 3, 4, 5}), *P* is the target detection accuracy (0 < *P* < 1), and *T* is the average time (sec) for a selection (*T* = 45). We used five kinds of natural sounds as auditory stimuli and enabled subjects to select five commands (*N* = 5). In addition to comparatively evaluating the detection performance of the natural sounds, we investigated the performance for fewer target candidates (*N* = {2, 3, 4}). As indicated in section 2.5, the LDA learns the target and non-target features and predicts probabilities for testing data. The probabilities were compared among five grand-average waveforms with five sound labels. One of the waveforms that indicated the highest probability was detected as an ERP waveform. Reducing the number of commands (e.g., five to three) may lead to an improved detection accuracy because the amount of misclassified data is decreased. Although the ITR gets worse by reducing the number of commands, accuracy is also important. We therefore evaluated the detection accuracy with five, four, three, and two commands. Training data were used in 1,650 sub-trials (11 trials × 30 sequences × 5 sub-trials), 1,200 sub-trials (8 trials × 30 sequences × 5 sub-trials), and 750 sub-trials (f trials × 30 sequences × 5 sub-trials) in each evaluation for four, three, and two commands, respectively. Four, three, and two grand-average ERP/non-ERP waveforms were the testing data in each evaluation. We assessed all combinations of the targets and showed the average of the results in the offline analysis. To complement the analysis, we evaluated the four, three, and two-command accuracies with training on all data; test data were the same as in the evaluation for the five commands. Non-candidates for classification output should not be included in the training data for cross-validation; however, they include two-class (ERP and non-ERP) information. The 2,100 sub-trials (14 trials × 30 sequences × 5 sub-trials) were used for training data in the evaluation for each four, three, and two commands. Four, three, and two grand-average ERP/non-ERP waveforms were used for the testing data.

Note that the first online experiment was sequentially conducted after the offline experiment; we therefore could not know the best combination of the natural sounds. We simply selected the target combination based on the sound directions. For a comparison with the first online experiment, we conducted a second online experiment under the same conditions.

## 3. Results

### 3.1. Detection Performances

To evaluate the performance of our auditory ERP-based BCI paradigm with a portable EEG device, we calculated an average detection accuracy and ITR through a LOTOCV procedure for each subject. In total, for each subject, 450 target epochs and 1,800 non-target epochs were recorded throughout the offline experiments. The detection accuracy and ITR are listed in Table 1.

**Table 1:** Summary of the detection performances in the offline analysis.

**Sub**.	**Five commands**		**Four commands**		**Three commands**		**Two commands**	
	**Accuracy**	**ITR (bits/min)**	**Accuracy (%)**	**ITR (bits/min)**	**Accuracy (%)**	**ITR (bits/min)**	**Accuracy (%)**	**ITR (bits/min)**
1	53.3	0.52	45.0	0.18	51.1	0.13	66.7	0.11
2	66.7	0.98	63.3	0.63	66.7	0.44	70.0	0.16
3	53.3	0.52	58.3	0.48	61.1	0.31	71.7	0.19
4	66.7	0.98	63.3	0.63	67.8	0.47	75.0	0.25
5	73.3	1.27	78.3	1.20	80.0	0.88	85.0	0.52
6	60.0	0.73	58.3	0.48	58.9	0.26	71.7	0.19
7	100	3.10	96.67	2.32	100	2.11	100	1.33
8	66.7	0.98	65.0	0.68	63.3	0.36	71.7	0.19
9	60.0	0.73	65.0	0.68	64.4	0.39	85.0	0.52
10	100	3.10	95.0	2.18	95.6	1.70	93.3	0.86
Mean	70.0	1.29	68.8	0.95	70.9	0.71	79.0	0.43
SD	16.1	0.93	15.6	0.70	15.2	0.63	10.6	0.37

*The average results of all target combinations were presented for <5 commands. Non-candidates for classification output were not included for the learning*.

First, we calculated the performance for five commands. As mentioned in section 2.6, LOTOCV was applied to 2,250 epochs. In each validation step, there were five testing waveforms (averaged across 30 epochs) containing one target, and four non-target data and 2,100 training epochs. This procedure yielded an average detection accuracy of 70.0% and an ITR of 1.29 bits/min. The accuracy for three out of the ten subjects was over 70.0% and the standard deviation of all subjects was 16.1%. In addition, both subjects 7 (healthy subject) and 10 (ALS patient) displayed high performance levels with a detection accuracy of 100% and ITRs of 3.10 bits/min.

Table 2 shows the confusion matrix with five sound labels in the offline analyses. The vertical labels indicate the true target sound labels. The horizontal labels are the predicted target sounds. The detection performance for sound “duck” was the lowest with an accuracy of 56.7%. The highest accuracy was 80.0%, attained by sound “singing bird” and “dove.” The highest false positive rate (FPR) was obtained by sound “duck,” for which six instances of “seagull” test data were misclassified to the sound across all subjects.

**Table 2:** Confusion matrix of five sound labels over all subjects in offline analysis.

		**Predicted labels**					**Accuracy (%)**
		**Duck**	**Bird**	**Frog**	**Seagull**	**Dove**	
True labels
	Duck	17	5	5	1	2	56.7
	Bird	2	24	1	1	2	80.0
	Frog	2	2	22	1	3	73.3
	Seagull	6	2	3	18	1	60.0
	Dove	0	3	2	1	24	80.0
Mean							70.0

Second, we decreased the number of commands from five to less than five to assess the viability of our paradigm using other output settings. When the number of commands was four, the average accuracy and ITR were 68.8% and 0.95 bits/min, respectively. With this setting, three of the ten subjects displayed over 70.0% detection accuracy. Three-command setting showed an average accuracy of 70.9% and ITR of 0.71. Three out of the ten subjects displayed over 70.0% accuracy. For two commands, the accuracy was 79.0% and the ITR was 0.43. With this setting, nine subjects displayed over 70.0% detection accuracy.

Third, we evaluated the detection performance when non-candidates for classification output were included in the learning. Table 3 shows the accuracies and ITRs. When the number of commands was four, the average accuracy and ITR were 73.8% and 1.16 bits/min, respectively. With this setting, six of the ten subjects displayed over 70.0% detection accuracy. Three-command setting showed an average accuracy of 78.7% and ITR of 0.95. Seven out of the ten subjects displayed over 70.0% accuracy. For two commands, the accuracy was 85.7% and the ITR was 0.63. With this setting, all ten subjects displayed over 70.0% detection accuracy. Unlike in ordinary LOTOCV, using non-candidates for classification output for learning may improve the detection performance.

**Table 3:** Detection performance when non-candidates for classification output were included in the learning.

**Sub**.	**Four commands**		**Three commands**		**Two commands**	
	**Accuracy (%)**	**ITR (bits/min)**	**Accuracy (%)**	**ITR (bits/min)**	**Accuracy (%)**	**ITR (bits/min)**
1	60.0	0.53	66.7	0.44	75.0	0.25
2	70.0	0.86	74.4	0.68	83.3	0.47
3	60.0	0.53	67.8	0.47	78.3	0.33
4	70.0	0.86	75.6	0.72	83.3	0.47
5	78.3	1.20	84.4	1.07	91.7	0.78
6	61.7	0.58	66.7	0.44	75.0	0.25
7	100	2.67	100	2.11	100	1.30
8	71.7	0.92	76.7	0.76	85.0	0.52
9	66.7	0.74	74.4	0.68	85.0	0.52
10	100	2.67	100	2.11	100	1.30
Mean	73.8	1.16	78.7	0.95	85.7	0.63
SD	14.2	0.78	11.8	0.61	8.6	0.38

*The average results of all target combinations were presented for <5 commands*.

In the online analyses, we evaluated the detection performance for three commands. For online experiment 1, we evaluated two runs performed in the same day as the offline experiment. The online detection accuracy and ITR are shown in Table 4. In total, 180 target and 720 non-target epochs were recorded for each subject. The average of the detection accuracy and ITR across all subjects was 80.0% and 1.16 bits/min. Under this setting, four out of ten subjects achieved 100% accuracy. In the runner-up, two out of the remaining six subjects, one of which was the ALS patient, had an accuracy of 83.3%. Table 5 shows the confusion matrix for the online three-command operation, which was composed of the sounds: duck, frog, and dove. The accuracy for the sound “frog” was the lowest. By contrast, the sound “duck” was correctly classified with an accuracy of 90.0%. Furthermore, we evaluated eight runs performed in online experiment 2. The online detection accuracy and ITR are shown in Table 4. In total, 900 target and 3,600 non-target epochs were recorded for each subject. The average of the detection accuracy and ITR across all subjects was 62.5% and 0.43 bits/min, respectively. Under this setting, three out of ten subjects achieved 70.0% accuracy. In addition, the ALS patient achieved an accuracy of 87.5%. Table 5 shows the confusion matrix. The accuracy for the sound “frog” was the lowest, as in online analysis 1. The sound “dove,” by contrast, was correctly classified with an accuracy of 66.3%.

**Table 4:** Summary of detection performances in online analyses for which the average accuracy of online analysis 1 was 80.0% with an ITR of 1.16 bits/min and that of online analysis 2 was 60.0% with an ITR of 0.44 bits/min.

	**Online analysis 1**		**Online analysis 2**	
**Sub**.	**Accuracy (%)**	**ITR (bits/min)**	**Accuracy (%)**	**ITR (bits/min)**
1	83.3	1.02	66.7	0.44
2	50.0	0.11	66.7	0.44
3	66.7	0.44	37.5	0.01
4	100	2.11	62.5	0.34
5	100	2.11	79.2	0.85
6	100	2.11	54.2	0.18
7	100	2.11	45.8	0.06
8	66.7	0.44	54.2	0.18
9	50.0	0.11	70.8	0.56
10	83.3	1.02	87.5	1.22
Mean	80.0	1.16	62.5	0.43
SD	19.4	0.83	14.3	0.36

**Table 5:** Confusion matrix of three sound labels over all subjects in online analysis.

**Online analysis 1**					
		**Predicted labels**			**Accuracy (%)**
		**Duck**	**Frog**	**Dove**	
True labels
	Duck	18	1	1	90.0
	Frog	3	13	4	65.0
	Dove	1	2	17	85.0
Mean					80.0
**Online analysis 2**					
		**Predicted labels**			**Accuracy (%)**
		**Duck**	**Frog**	**Dove**	
True labels
	Duck	50	13	17	62.5
	Frog	19	47	14	58.8
	Dove	18	9	53	66.3
Mean					62.5

### 3.2. ERP Analysis

**Figure 3:**
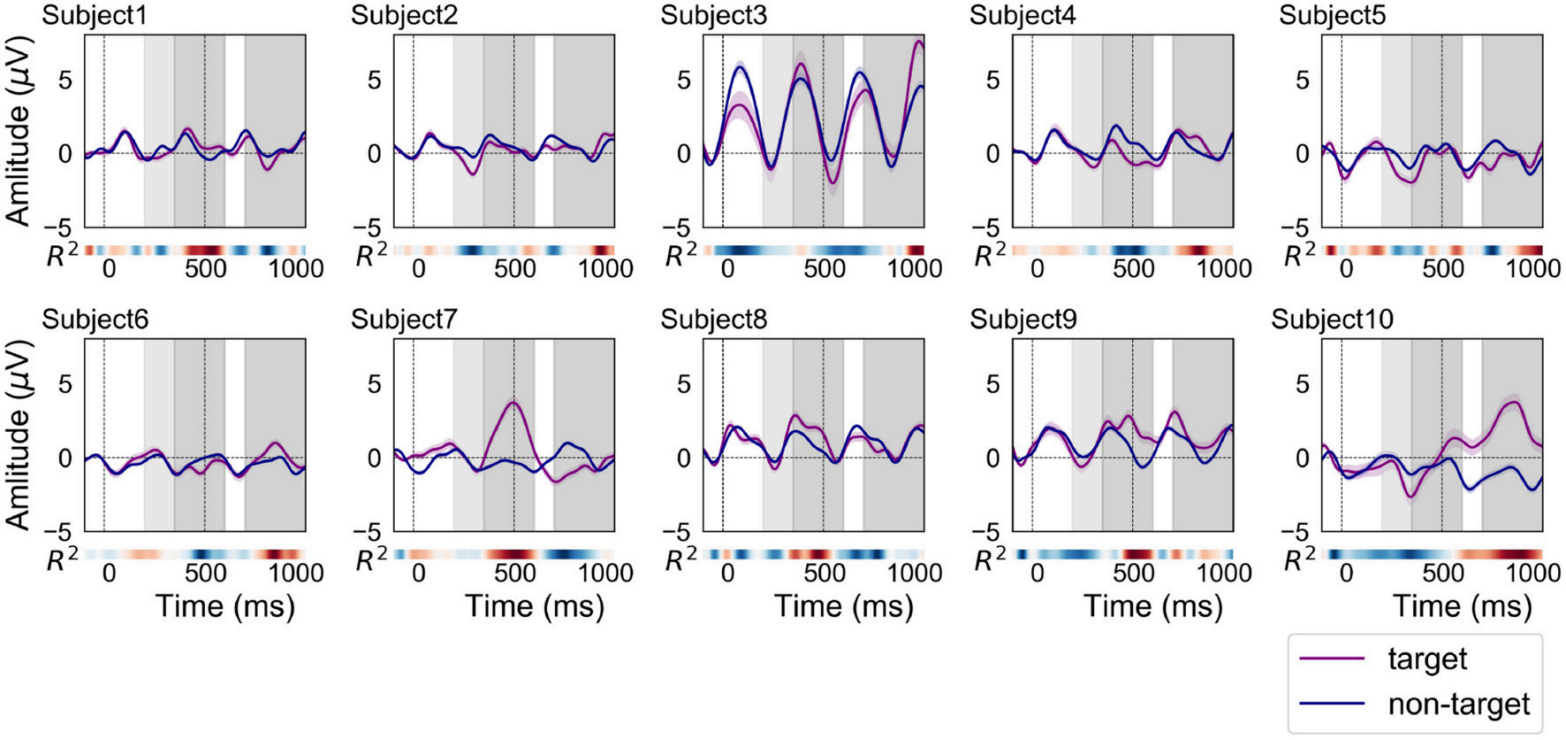
ERP and non-ERP waveforms of each subject in the offline analysis (purple line: ERP, blue line: non-ERP). The filled fields along the plots denote the standard error (SE) of amplitudes for each time point. The time interval for N200 is filled with light-gray color at 200–350 ms. In addition, the time intervals between 350–600 and 700–1,000 ms were filled with dark-gray color for finding the positive peaks of ERP responses.

Figure 3 shows the grand average of ERP and non-ERP waveforms for each subject in the offline analysis. In total, 450 target and 1,800 non-target epochs were recorded per subject over the course of these experiments. The filled fields along the plots denote the standard error (SE) of amplitude for each time point. Typically, the meaningful components of ERP-based BCI are N200 and P300 (Höhne et al., 2011; Hübner et al., 2018). The N200 components were uniquely and visually identified around the light-gray fields at 200–350 ms in Figure 3. A heat map in the form of a colored bar is positioned beneath each ERP plot to indicate the signed *R*^2^ value. The value specifies how clearly the ERP and non-ERP are discriminated. The discriminability is described by the heat maps. The colors in the heat maps indicate the strength of *R*^2^. Through the *R*^2^ plot, the highest positive amplitudes within the target epochs could be identified on two-time segments positioned at ~350–600 and 700–1,000 ms ranges. These areas are visually distinguished by dark-gray shading in Figure 3.

**Figure 4:**
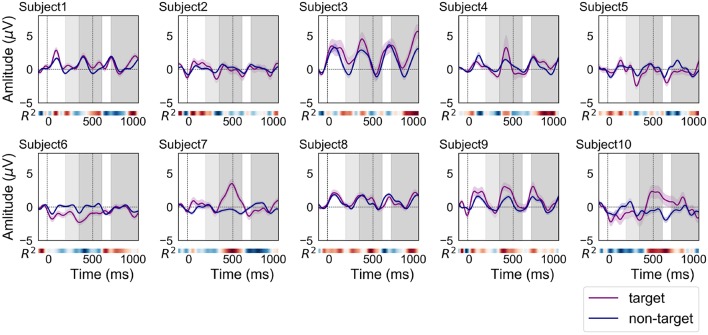
ERP and non-ERP waveforms of each subject in the online analysis 1 (purple line: ERP, blue line: non-ERP). The filled fields along the plots denote the standard error (SE) of amplitudes for each time point. The time interval for N200 is filled with light-gray color at 200–350 ms. In addition, the time intervals between 350–600 and 700–1,000 ms were filled with dark-gray color for finding the positive peaks of ERP responses.

Figure 4 shows the grand average of ERP and non-ERP waveforms for each subject in the online analysis 1. In total, 180 target and 720 non-target epochs were recorded per subject over the course of the experiments. Most of the ERP waveforms were similar in shape and size to those of the offline analysis. A notable difference in comparison to the waveforms from the offline experiments was that the discriminability time points as well as their strengths differed. The red color, which indicates a high *R*^2^ value, can be observed to deepen around 400 ms for subjects 3 and 4. For subjects 6, 9, and 10, higher *R*^2^ values appeared earlier than in the offline experiment.

**Figure 5:**
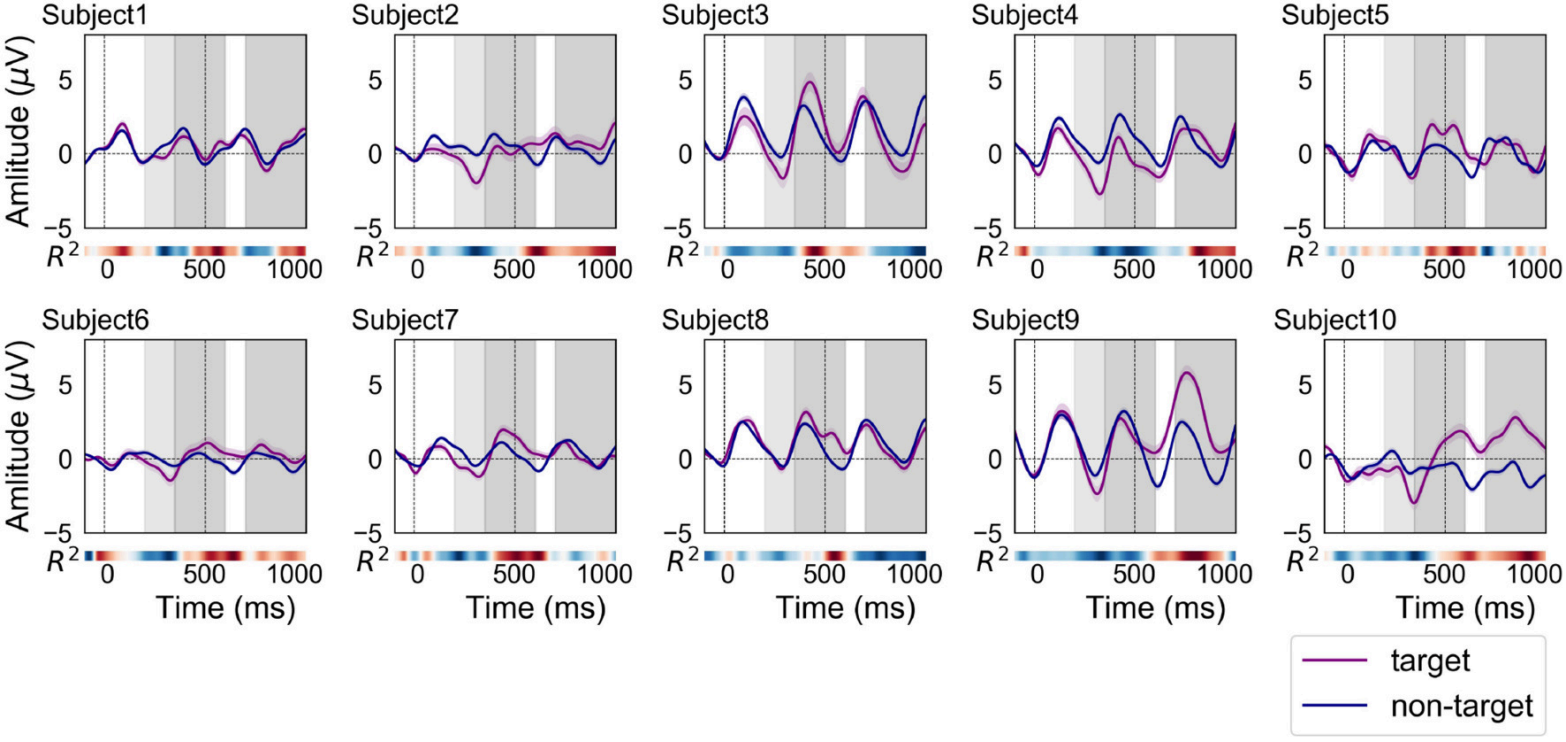
ERP and non-ERP waveforms of each subject in the online analysis 2 (purple line: ERP, blue line: non-ERP). The filled fields along the plots denote the standard error (SE) of amplitudes for each time point. The time interval for N200 is filled with light-gray color at 200—350 ms. In addition, the time intervals between 350–600 and 700–1,000 ms were filled with dark-gray color for finding the positive peaks of ERP responses.

Figure 5 shows the grand average of ERP and non-ERP waveforms for each subject in the online analysis 2. In total, 900 target and 3,600 non-target epochs were recorded over the experiments of each subject. Most of personal characteristics of the ERP and non-ERP waveforms were not significantly different from those of online experiment 1. However, the time points representing strong *R*^2^ values were moved to forward (subjects 3, 5, and 6) and backward (subjects 9 and 10).

Table 6 displays the latency and amplitude of negative and positive peaks in ERP waveforms from the offline experiments. The negative and positive peaks were found within the intervals of 200–350 and 350–1,000 ms, respectively. There were two time segment candidates to appear as a positive peak in an ERP waveform. Usually, a positive peak (P300) should appear in the 350-600 ms range, but sometimes they appeared in the 700–1,000 ms range in this study. The ERP waveform of subject 7, which has contained the most discriminable information in the offline analysis 1 (see Table 1), had a strong positive peak at ~500 ms. Subject 10, which has also contained the most discriminable information in the offline analysis 1, had a positive peak after 700 ms. In online analysis 2, the average of latencies of positive peaks were decreased compared to offline analysis and online analysis 1. Although the latencies for some subjects were increased, the tendency as a whole subject indicates that it is shortened.

**Table 6:** Overview of latencies and amplitudes of positive (200–350 ms) and negative (350–1,000 ms) ERP peaks in the offline and online analyses.

	**Offline**				**Online 1**				**Online 2**			
	**Negative**		**Positive**		**Negative**		**Positive**		**Negative**		**Positive**	
**Sub.(ms)**	**Lat. (ms)**	**Amp. (μV)**	**Lat**.	**Amp. (μV)**	**Lat. (ms)**	**Amp. (μV)**	**Lat. (ms)**	**Amp. (μV)**	**Lat. (ms)**	**Amp. (μV)**	**Lat. (ms)**	**Amp. (μV)**
1	281	−0.51	537	0.08	324	−0.22	941	1.34	287	−0.59	777	1.25
2	293	−1.79	932	0.68	340	−1.45	977	0.58	347	−0.74	799	1.37
3	199	−1.42	965	5.95	313	1.23	986	4.90	313	−0.86	525	4.82
4	299	−1.56	828	0.46	338	−1.62	932	−0.28	347	−0.76	844	1.68
5	279	−1.49	998	0.70	313	−2.58	998	1.03	347	0.15	535	1.96
6	314	−0.12	844	1.20	320	−1.67	641	−0.30	309	−0.51	611	1.06
7	309	−1.16	508	3.40	295	−0.87	494	3.41	340	−0.62	541	1.95
8	260	−1.33	473	1.43	205	−0.32	475	0.89	347	−0.06	506	3.14
9	240	−1.36	500	1.75	299	−1.27	395	2.64	324	−0.44	855	5.78
10	340	−2.74	895	3.15	244	−0.90	635	0.90	220	−1.08	949	2.78
Mean	281	−1.35	748	1.88	299	−0.97	747	1.51	329	−0.55	694	2.58
SD	38	0.67	205	1.71	41	0.98	230	1.57	40	0.35	158	1.51

## 4. Discussion

As mentioned earlier, the aim of this study is to analyze the discriminability of auditory ERP waveforms acquired from a consumer-grade single-channel EEG device under offline and online conditions. Our results indicate that the single-channel EEG device can be feasibly used for both offline and online analyses.

The offline analysis revealed that the detection accuracy and ITR for five, four, three, and two commands averaged across 10 subjects were 70.0% and 1.29 bits/min, 73.8% and 1.16 bits/min, 78.7% and 0.95 bits/min, and 85.7% and 0.63 bits/min, respectively. The online analysis confirmed that the detection accuracy and ITR for three commands averaged across 10 subjects were 80.0% and 1.16 bits/min. In this condition, four out of the 10 subjects achieved 100% accuracy (2.11 bits/min). In addition to detection performance, we investigated the shape of ERP waveforms. The ERP analysis revealed that N200 and P300 components can be detected even if we obtained prefrontal single-channel EEG data. Additionally, the discriminability of ERP waveforms was clarified by signed *R*^2^ values.

### 4.1. Experimental Design

To compare auditory ERP detection performance using a single-channel prefrontal EEG to multichannel EEGs, we designed an experimental protocol with a set of natural sounds identical to those utilized in a previous study that made use of multichannel data from a traditional EEG device (Simon et al., 2015). In our protocol, the duration of the ISI and the number of sequences were modified from their initial settings (250 ms and 10) to 150 ms and 30, respectively, because they did not contribute to achieving high detection performance in our pre-test trials, where three participants joined. While we set the duration of the ISI and the number of sequences to 250 ms and 10 sequences, when the length of sound presentation was 150 ms, the average accuracy was 40.0 ± 5.4%. Consequently, we increased the number of sequences from 10 to 30; however, it took 60 s to complete each trial, which induced fatigue and boredom in the users, thereby decreasing the average accuracy. When testing 150 ms ISI with 10 sequences, the accuracy was 46.7 ± 33.9%. Finally, we shortened the ISI from 250 ms to 150 ms with 30 sequences and acquired 75.6 ± 19.1%. Therefore, the balance between the number of sequences and the trial length was important. The most important factor influencing our experimental design was the subject's motivation (Nijboer et al., 2008; Kleih et al., 2010). Fatigue and boredom decrease the motivation of subjects and results in an overall decline in performance during the auditory ERP-based BCI. In consideration of the negative consequence of these emotions, setting the ISI to a duration of 150 ms was empirically the best solution. Although the duration must be optimized based on a quantitative and strict approach in future studies, to prevent fatigue or boredom, we found that a trial with less than or equal to 45 s does not tend to induce such feelings. Therefore, we set the ISI period to 150 ms and the number of sequences to 30 in the offline analysis.

**Figure 6:**
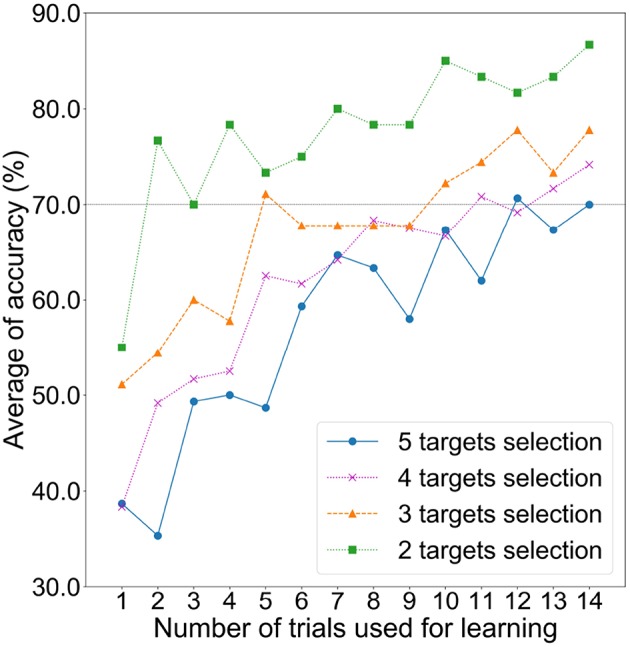
Average detection accuracies based on LOTOCV for increasing the number of learning trials with two, three, four, and five commands.

To assess the relationship between the number of trials and the detection accuracy, we investigated the detection accuracy with an incremental learning approach. Figure 6 shows the change in accuracy when the number of trials for learning was sequentially increased from one to 14. Here, one trial data was always employed as testing data in a LOTOCV procedure. The learning trial was randomly chosen. Figure 6 shows an upward trend for average accuracy regardless of the number of commands. For this result, the accuracy could be improved when obtaining more trials. However, the purpose of this study is to evaluate the feasibility of auditory ERP-based BCI with prefrontal single-channel EEG data. Exceeding 70.0% accuracy, which is required for satisfactory communication in BCI operation (Käthner et al., 2013), is a reasonable criterion for confirmation of the feasibility. As we estimated above, the average accuracy for five commands exceeded this criterion when the number of learning trials was set to 12. Therefore, we assumed that 15 trials for each subject was an adequate number of trials.

The online experiments were designed to achieve the optimal balance between the detection performance and the subject motivation. We chose three natural sounds (duck, frog, and dove) for the following two reasons. First, it was necessary to reduce the number of targets to maintain an acceptable detection accuracy (over 70.0%) because subjects' motivation will decrease if they experience difficulty in learning the auditory ERP-based BCI system. In our pre-test trials, subjects tended to get tired when five-command operation was conducted in the online experiments, because they hardly selected the true targets. Hence, we decreased the number of the target sounds and attempted to increase the accuracy. The second reason is to keep in check the total experiment duration. To avoid any changes in device configuration that may result from reattachment and lead to deterioration of the constructed classifiers, we continuously recorded and tested EEG data under offline and first online conditions for ~1 h. Although we allowed subjects to take a break between runs, they tended to experience fatigue when the EEG recording continued for more than 1 h. Considering these reasons, we decided to conduct two runs with three commands in the online experiment 1.

### 4.2. BCI Performance

Our measurement configuration differed from that of previous studies—which have typically positioned electrodes at vertices around the temporal and centro-parietal region, such as Cz and Pz positions—in that our electrode was positioned at the Fpz position, directly over the prefrontal region. Despite this difference, the performance of our experiments was not much lower than what previous studies have reported using either multi- or single-channel methods. The average detection accuracy in offline experiment and online experiment 1, as described in section 3.1, exceeded the criteria of 70.0%. The average accuracy of online experiment 2 was 62.5% whereas the date of the experiment was much later than that of the first experiments. Höhne et al. (2011) previously reported a detection accuracy of 89.4% and an ITR of 3.40 bits/min when using nine auditory stimuli in an online analysis that made use of multichannel data with a traditional EEG device. Simon et al. (2015) demonstrated that performance during online five-command operation for auditory ERP-based BCI using multichannel EEGs can yield a detection accuracy of 90.0% (ITR of 4.23 bits/min) for 11 healthy subjects and 45.0% (ITR of 1.35 bits/min) for an ALS patient. Furthermore, Lopez-Gordo et al. (2012) reported that using single-channel EEG data from position Cz and speech sounds as stimuli for auditory ERP-based BCI obtained 69.0–78.0% detection accuracy with an ITR of 2.70 bits/min. In our study, the averaged detection accuracy across 10 subjects in the offline five-command operation was 70.0% and the ITR was 1.29 bits/min. The average accuracy of the first online three-command operation was 80.0% and the ITR was 1.16 bits/min. In addition, subjects 7 and 10 displayed high-performance levels with detection accuracy for five, four, three, and two commands in the offline analysis. Interestingly, the ALS patient (subject 10) demonstrated the maximum performance. The subjects showed 100 and 83.3% in the online experiment, whereas the ALS patient showed the second highest accuracy. These results indicate that the BCI paradigm would not be limited to healthy subjects. For the detection accuracy, it was confirmed that our paradigm maintained a high level of accuracy without using any vertex channels.

From Tables 1, 3, the average accuracies for four, three, and two command conditions were improved while using non-candidate data as learning data. In the LOTOCV, all trials should be testing data. Table 1 indicated the result in an ordinary case. However, the non-candidate data for testing also has information of ERP or non-ERP. Therefore, we added the non-candidate data for learning data and obtained higher accuracies. At this time, the number of training samples was equal for all command conditions. The results indicate that increasing the amount of data would be useful even if the data contain information unrelated to the candidate sounds.

For long-term use of our BCI paradigm, the result of online analysis 2 is important. The average accuracy was 62.5% and did not exceed 70.0%; however, the results of our research are valuable for future studies. First, the result showed that three out of ten subjects achieved 70.0% accuracies. They indicated the possibility that our BCI paradigm can be used as a communication tool even if a limited number of persons can use it. Second, the accuracies of subjects 2, 9, and 10 in online analysis 2 were higher than those in online analysis 1. Therefore, the long-term use is not a factor that decreases the accuracy. Third, the accuracy of subject 10 (ALS patient) showed the highest accuracy in online analysis 2. More ALS patients are needed to show that this BCI paradigm can be used for the patients, but it is a fact that the ALS patient showed a sufficient accuracy in long-term use. In addition, the time point where a strong *R*^2^ value appears moved forward (subjects 3, 5, and 6) and backward (subjects 9 and 10) in online analysis 2. This is because the offline data and online data 2 were obtained on a different day, and a reattachment of the EEG device has occurred. Therefore, solving these issues by the domain adaptation or transfer learning may improve the accuracy in future work.

After the online experiment 2, we obtained an interesting comment from subject 5 whose accuracy of the online analysis 2 was 79.2%. Even before the system showed the result, he could notice whether he correctly selected the target command. He said that the point was to hear non-target sounds as a noise. When he inevitably heard those sounds as a natural sound, the system showed a wrong command. The comment may improve accuracy of the BCI by explaining this fact before the auditory BCI experiments.

De Vos et al. (2014) conducted a three-command auditory oddball task using a low-cost, small, and wireless 14-channel EEG measurement configuration for auditory ERP-based BCI in daily life. They obtained a detection accuracy of 71.0% with an ITR of 1.06 bits/min. In comparison to this study, our BCI framework of offline analysis and online analysis 1 yielded a better accuracy and ITR while making use of a low-cost, small, and wireless single-channel EEG measurement configuration. Moreover, the preparation time for using a wearable device is <1 min. When the preparation of the EEG device is considered, our framework is faster than those reported in previous studies. The study of auditory ERP-based BCI using prefrontal single-channel EEG is still in its infancy; however, this study succeeded in maintaining an adequate level of accuracy and encourages daily-use portable auditory ERP-based BCIs in daily life environments. The next challenge is to improve ITRs.

### 4.3. ERPs Acquired From a Prefrontal Single-Channel EEG Device

Although the latency of positive peaks varied across subjects (SD of offline = 205 ms, online 1 = 230 ms, online 2 = 158 ms), the BCI performance maintained a high level of accuracy in all subjects (see Table 1). Figures 3–5 indicate that some ERP waveforms had positive peaks at ~350–600 ms time segment, which is in line with the findings reported in previous studies of auditory ERP-based BCIs (Schreuder et al., 2010; Höhne et al., 2011; Simon et al., 2015). On the other hand, some ERP waveforms had positive peaks at ~700–1,000 ms segment. The appearance of positive peaks after 700 ms from stimulus onset was not explicitly discussed in previous studies; however, they were frequently confirmed in auditory ERP-based BCI studies (Furdea et al., 2009; Höhne and Tangermann, 2014). They also showed that the positive peak after 700 ms contributes to auditory BCI accuracy. The results did not detect any new neuronal phenomena, but merely suggest that the delay of negative and positive peaks (i.e., N200 and P300) may be caused by the difficulty in identifying auditory stimuli. Therefore, detecting P300 is not the only key factor for obtaining sufficient auditory ERP-based BCI performance.

The relationship between ERP and non-ERP waveforms of subject 3 was different than those of our other subjects. The amplitudes of both waveforms were much larger than those in the other plots. The first online accuracy of subject 3 was low, only 66.7%; however, this result was not significantly lower than that of the other subjects. The accuracy in the offline analysis for two commands was 85.7% (Table 1). This result demonstrates that sufficient accuracy can be achieved even though the ERP waveforms do not show typical P300 components. The result of online analysis 2 was low for subject 3; this is because the latency of the positive peak moved from 986 ms to 525 ms. Most of the previous studies showed that the peak appears at approximately 250–600 ms (Furdea et al., 2009; Höhne and Tangermann, 2014). Therefore, the subject might have to get used to the BCI paradigm, and obtaining learning data on the same day as the long-term testing would improve accuracy.

A critical point in our examination of auditory ERP-based BCI is the similarities between the evoked ERPs over subjects. The latencies of some subjects in online analyses were lower when compared to the offline analysis. These variations result from the users becoming accustomed to hearing sounds or visual feedback. Previous studies revealed that repeated training could generate similar ERP waveforms in the subject (Baykara et al., 2016; Halder et al., 2016). Although we only conducted six trials in the online experiment 1 to maintain a high level of subject motivation, future collection of more data related to training and visual feedback would allow us to evaluate whether or not repetitive training improves the performance of auditory ERP-based BCI with prefrontal single-channel data.

Erlbeck et al. (2017) showed that there was no difference in auditory P300 between ALS patients and healthy subjects. In this study, similar ERP waveforms could be obtained from ALS patient in both offline and online experiments. While it is difficult to conclude that there is no difference between ERPs of healthy subjects and ALS patients because only one ALS patient participated in our study, our results indicate that the user with ALS was able to operate the auditory ERP-based BCI with a prefrontal single-channel EEG device in a manner comparable to that of healthy users.

### 4.4. Limitations

In this study, all subjects including an ALS patient yielded good BCI performance. It should be noted that the participants in our study were already accustomed to wearing an EEG device. The ALS patient had taken part in other EEG projects and had a good understanding of the EEG device. The other subjects were researchers and engineers in our institution; thus, they feel no resistance to EEG measurements. In addition, the ALS patient was a musician, which may have contributed to his ease in distinguishing the sounds. The ability to discriminate between sounds has been demonstrated to differ between musicians and non-musicians (Paraskevopoulos et al., 2014).

As mentioned in section 2, the experiments were conducted while subjects had their eyes closed, except for when checking the feedbacked number in the online analysis. Prefrontal EEGs in all cases contain eye blink and eye movement artifacts when subjects have their eyes open. As a result, the findings of this study are only applicable to subject in the eyes-closed state or ALS patients whose eye blink ability has been lost. To analyze an auditory ERP-based BCI using prefrontal EEG data in an eyes-open state, an ocular artifact reduction method would need to be applied to the recorded EEG data.

We obtained a detection accuracy of 80.0% using three target sounds in online analysis 1; however, the ITR was only 1.16 bits/min. Most previous studies have developed auditory ERP-based BCIs to control some effectors such as virtual keyboard as movement-independent communication tools (Schreuder et al., 2010; Höhne et al., 2011; Simon et al., 2015). These studies attained ITRs of over 2.00 bits/min. The lower ITR yielded by our procedure is not appropriate for the development of such tools. At present, single-channel EEG devices should be reserved for different purposes compared to multichannel devices, as dictated by the situation.

Moreover, in online analysis 2, the detection performance was totally decreased. Data adaptation approaches for modifying the effects of electrode shifts and instrument changes must be applied in trained models to implement more effective portable BCIs in everyday life. Our study was the first to analyze the performance of auditory ERP-based BCI based on prefrontal single-channel data obtained from a consumer-grade wireless EEG device in offline and online conditions. These results would help provide easy-to-use ERP-based BCIs to ALS patients in their home environments.

## 5. Conclusions

The development of a portable auditory ERP-based BCI would help overcoming considerable obstacles preventing the use of BCI technology in everyday life. In this study, we analyzed prefrontal single-channel EEG data obtained from a consumer-grade EEG device using a natural sound-based auditory oddball paradigm. The offline and online analyses demonstrated sufficient accuracy for both healthy subjects and an ALS patient. The offline analysis indicated that the detection accuracy and ITR averaged across ten subjects for five, four, three, and two commands were 70.0% and 1.29 bits/min, 73.8% and 1.16 bits/min, 78.7% and 0.95 bits/min, and 85.7% and 0.63 bits/min, respectively. The online analysis 1 indicated that the detection accuracy and ITR averaged across all subjects for three commands were 80.0% and 1.16 bits/min. Online analysis 2, which obtained data on a different day than the offline analysis and online analysis 1, showed that the detection accuracy and ITR were 62.5% and 0.43 bits/min, respectively. Whereas the ITRs were lower than in previous studies using clinical multichannel EEG device, our paradigm with a single-channel EEG device maintains the accuracy required for sufficient communication and has extreme portability for daily use. This study represented the feasibility of prefrontal single-channel EEGs for auditory ERP-based BCIs. The improvement of accuracy by training more trials, and the effects of fatigue caused by continuous online experiments remain issues that should be evaluated in further studies.

## Ethics Statement

We provided the complete ethics statement in the manuscript as follows: The experimental design was approved by the Ethical Review Committee of Dentsu ScienceJam Inc. with approval number 006 in October 2018. All of the participants provided written informed consent.

## Author Contributions

MO and MM had the initial idea for this study and performed the data collection. MO and SK contributed to the data analyses and the interpretation of the results. MO completed the first draft of the article. All authors participated in revising the draft to finalize the manuscript and contributed to the planning and design of the study.

### Conflict of Interest Statement

MO is an employee of Dentsu ScienceJam Inc., Tokyo, Japan. The EEG devices (MindWave Mobile) used in this study were provided by Dentsu ScienceJam Inc. The place of the experiments for healthy subjects was conducted at an office of Dentsu ScienceJam Inc. The experimental design was approved by the Ethical Committee of Dentsu ScinceJam Inc. The remaining authors declare that the research was conducted in the absence of any commercial or financial relationships that could be construed as a potential conflict of interest.
